# A Matched Comparison Across Three Different Sensory Pairs of Cross-Modal Temporal Recalibration From Sustained and Transient Adaptation

**DOI:** 10.1177/2041669517718697

**Published:** 2017-07-05

**Authors:** David Alais, Tam Ho, Shui’er Han, Erik Van der Burg

**Affiliations:** School of Psychology, The University of Sydney, Australia; School of Psychology, The University of Sydney, Australia; Department of Experimental and Applied Psychology, Vrije Universiteit Amsterdam, The Netherlands

**Keywords:** temporal recalibration, perceptual synchrony, sequential dependency, adaptation, audiovisual, visuotactile, audiotactile

## Abstract

Sustained exposure to an asynchronous multisensory signal causes perceived simultaneity to shift in the direction of the leading component of the adapting stimulus. This is known as temporal recalibration, and recent evidence suggests that it can occur very rapidly, even after a single asynchronous audiovisual (AV) stimulus. However, this form of rapid recalibration appears to be unique to AV stimuli, in contrast to recalibration following sustained asynchronies which occurs with audiotactile (AT) and visuotactile (VT) stimuli. This study examines temporal recalibration to AV, VT and AT asynchrony with spatially collocated stimuli using a design that produces both sustained and inter-trial recalibration by combining the traditional sustained adaptation approach with an inter-trial analysis of sequential dependencies in an extended test period. Thus, we compare temporal recalibration to both sustained and transient asynchrony in three crossmodal combinations using the same design, stimuli and observers. The results reveal that prolonged exposure to asynchrony produced equivalent temporal recalibration for all combinations: AV, AT and VT. The pattern for rapid, inter-trial recalibration was very different. Rapid recalibration occurred strongly for AV stimuli, weakly for AT and did not occur at all for VT. For all sensory pairings, recalibration from sustained asynchrony decayed to baseline during the test phase while inter-trial recalibration was present and stable throughout testing, suggesting different mechanisms may underlie adaptation at long and short timescales.

## Introduction

Living in a constantly changing environment requires sensory mechanisms capable of adapting flexibly to novel situations. This is a particular challenge in multisensory processing because the neural timing of signals does not directly reflect stimulus timing in the environment. Factors such as neural latencies, signal intensity, attention and source distance all affect the timing of neural responses, ensuring that external synchrony rarely produces neural synchrony (Alais, Newell, & Mamassian, 2010; Burr & Alais, 2006). Nonetheless, the perceptual system appears able to deal with misaligned multisensory inputs through an adaptation process known as temporal recalibration which effectively realigns multisensory signals, as demonstrated by a number of psychophysical studies (Fujisaki, Shimojo, Kashino, & Nishida, 2004; Navarra et al., 2005; Roseboom & Arnold, 2011; Vatakis, Navarra, Soto-Faraco, & Spence, 2007; Vroomen, Keetels, De Gelder, & Bertelson, 2004; Yarrow, Roseboom, & Arnold, 2011). These studies showed that several minutes of repeated exposure to an audiovisual (AV) stimulus with a fixed asynchrony (*adaptation phase*) causes a shift in perceived timing in a subsequent *test phase* such that the point of subjective simultaneity (PSS) shifts towards the leading sensory modality in the adaptation phase. This shift can be termed a recalibration in that it involves a lateral shift to realign subjective timing. It may play a functional role by compensating for changes in physical timing to maintain component signals in a narrow range where integration is more likely, or it may simply arise as a consequence of typical repulsion recalibration effects that are widely observed in sensory systems following sustained exposure to a stimulus (Linares, Cos, & Roseboom, 2016; Roseboom, Linares, & Nishida, 2015).

Sensory adaptation effects are well known in many domains and are usually demonstrated by behavioural, perceptual or neural changes that emerge after prolonged exposure to an adapting stimulus (Green & Bavelier, 2008; Stein & Rowland, 2011). There is emerging evidence, however, that adaptation to multisensory timing can occur over very short periods of time. For example, multisensory neurons in the superior colliculus have been shown to adapt quickly to asynchronous AV stimuli, as if realigning responses to match the input timing (Yu, Stein, & Rowland, 2009), and recent psychophysical findings by Van der Burg, Alais, and Cass (2013) demonstrate that recalibration can occur after exposure to a single asynchronous AV stimulus, especially when vision leads the auditory stimulus. Van der Burg et al.’s design was a novel one which removed the adaptation phase typical of recalibration experiments and subjected participants only to the test phase. In common with many recalibration studies, the test phase was a rapid series of trials involving AV signals with randomly varying stimulus onset asynchronies (SOAs), with the participant making a simultaneity judgement after each trial. Conventionally, responses are binned by SOA and a Gaussian simultaneity function is fitted. In Van der Burg et al.’s study, however, an inter-trial analysis was used which binned the current Trial (*t*) into one of two categories, determined by the modality order (A led or V led) on the previous Trial (*t–1*). Their analysis revealed that synchrony judgements on a given Trial (*t*) were drawn towards the leading modality on the previous Trial (*t–1*). This shows a recalibration shift dependent on a single AV asynchrony, and moreover, the size of this adaptation effect was surprisingly large and similar in magnitude to the shifts produced by sustained adaptation (Fujisaki et al., 2004; Vroomen et al., 2004). A sequential dependency between trials is not limited to AV timing and has been recently reported in audition (Alais, Orchard-Mills, & Van der Burg, 2015) and in various visual contexts (Alais, Leung, & Van der Burg, 2017; Cicchini, Anobile, & Burr, 2014; Fischer & Whitney, 2014; Liberman, Fischer, & Whitney, 2014; Taubert, Alais, & Burr, 2016; Taubert, Van der Burg, & Alais, 2016). In some cases, the dependency is consistent with a repulsive effect (as in Alais et al., 2015 and Van der Burg et al., 2013), and in others, it is attractive (Cicchini et al., 2014; Fischer & Whitney, 2014; Liberman et al., 2014; Taubert, Van der Burg, et al., 2016). It is even possible for both serial dependencies to be present simultaneously for different attributes of a stimulus (Alais et al., 2017; Taubert, Alais, et al., 2016).

The mechanism underlying rapid, inter-trial temporal recalibration appears to differ from that of conventional recalibration produced by prolonged adaptation. Whereas prolonged adaptation elicits recalibration shifts lasting for a minute or so, inter-trial recalibration is very short-lived and largely vanishes after a single trial because a given test trial both reveals the adaptation effect from the previous trial as well as inducing a new recalibration for the subsequent test trial. Apart from their different time-courses, there is another striking difference: recent results suggest that rapid temporal recalibration is unique to AV perception, as no one-back recalibration shifts were observed for audiotactile (AT) or visuotactile (VT) stimuli (Van der Burg, Orchard-Mills, & Alais, 2015). This suggests two possible conclusions, either that AT and VT rapid recalibration simply do not occur at all or perhaps if they do occur, they require prolonged adaptation. Previous recalibration studies using prolonged adaptation with AT and VT stimuli are inconclusive on this point as some report PSS shifts after prolonged exposure to asynchronous AT and VT stimuli (Hanson, Heron, & Whitaker, et al., 2008; Keetels & Vroomen, 2008; Takahashi, Saiki, & Watanabe, 2008), while others do not (Harrar & Harris, 2005, 2008). This discrepancy may be driven in part by differences in auditory presentation methods, as transfer of AV recalibration was observed in VT pairings when headphones were used, whereas collocated AV stimuli resulted in a transference to AT pairings (Di Luca, Machulla, & Ernst, 2009). These findings suggest a role for spatial collocation in VT and possibly AT recalibration, in contrast to AV temporal recalibration which occurs regardless of whether the sound is presented over headphones or collocated with the visual stimulus (Di Luca et al., 2009; Fujisaki et al., 2004).

In the present experiment, we will examine temporal recalibration of AV, VT and AT stimuli using a design based on Van der Burg, Alais, and Cass (2015) that will reveal the effects of both prolonged and inter-trial recalibration within a single experiment. The advantage of doing so is that it allows comparison across three modality pairings within a standardised design between prolonged and inter-trial adaptation, and in addition, we will do so using the same group of observers and collocated A, V and T stimulus components for all conditions. This will establish whether rapid recalibration is indeed absent for AT and VT stimuli on a trial-by-trial basis (Van der Burg, Orchard-Mills, et al., 2015) and also clarify that AT and VT recalibration do indeed occur following prolonged adaptation to collocated stimuli. If we do find AT and VT recalibration after prolonged adaptation but no inter-trial recalibration for the same stimulus pairings, this will support the conclusion that two distinct mechanisms underlie temporal recalibration from sustained and transient stimuli. Moreover, using a standardised design and stimuli on a common group of observers means that any failure to find recalibration in a particular condition cannot be attributed to differences in stimulus, procedures or subjects.

## Methods

### Participants

Twenty participants (13 females; mean age: 21.6, ranging from 18 to 25 years) with normal or corrected vision and reporting no hearing or neurological disorders were recruited for this study. All were naïve as to the purposes of the study and were paid $AU 20 per hour for their participation.

### Stimuli and Apparatus

**Figure 1.**
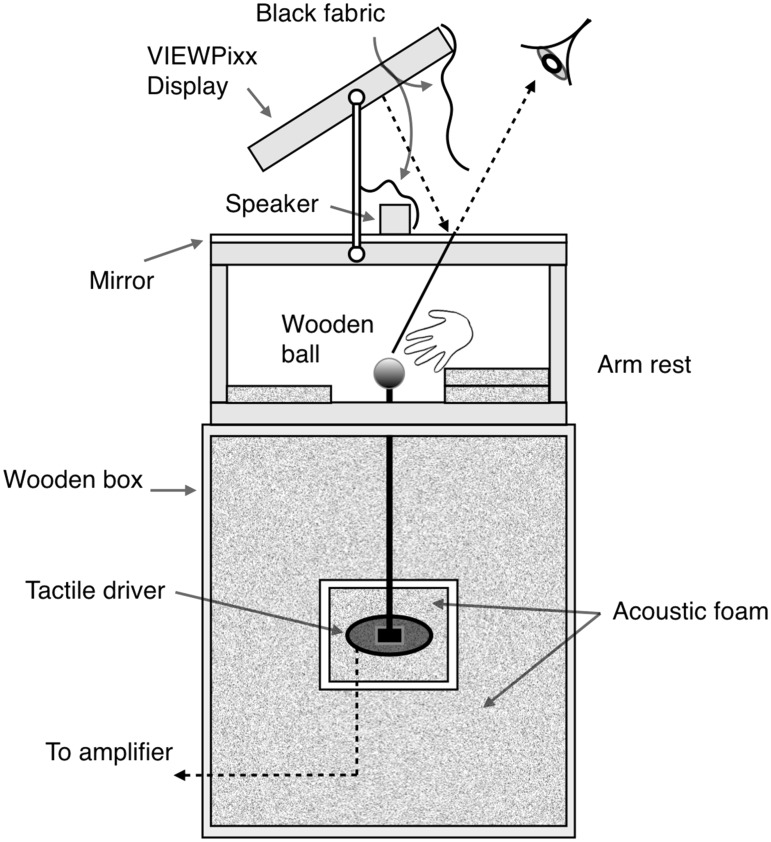
*Apparatus:* To spatially collocate the visual, auditory and tactile stimuli, the reflection of the visual stimulus was presented on a mirror 285 mm away from the eyes and 285 mm away from the VIEWPixx LCD display, making the optical path length equal to the distance from the top of the wooden ball to the eyes (570 mm). The auditory stimulus was presented with a small powered speaker located on the same plane as the visual stimulus’s reflection and adjacent to it. Both the wooden ball and speaker were hidden from view by the mirror and black fabric respectively.

The experiment took place in a dimly lit room. Stimulus presentation and response collection were controlled using MATLAB (Mathworks Inc, Natick, MA, USA) and the PsychophysicsToolbox extensions (Brainard, 1997; Pelli, 1997). The visual stimulus was a white ring (0.86° wide, 8.6° outer diameter, and luminance of 49.6 cd/m^2^) presented for 50 ms around a white fixation cross on a grey background (24.8 cd/m^2^). The tactile stimulus was a 150 Hz sinusoid with a root-mean-square intensity of 0.12 and the auditory stimulus was a 1 kHz tone with a root-mean-square intensity RMS of 0.17. The auditory and tactile stimuli had a sampling frequency of 44.1 kHz and were presented for 50 ms and were cosine ramped for 5 ms at onset and offset. As shown in Figure 1, the visual stimulus was presented on a VIEWPixx LCD display (resolution of 1920 × 1200; refresh rate 120 Hz; VPixx Technologies Inc, Sait-Bruno, QC, Canada) and reflected from a mirror to the observer to allow easy collocation with the tactile stimulus located beneath and making an optical path length (57 cm) matching the distance to the tactile stimulus. The tactile stimulus was a wooden ball of 62 mm diameter presented to the dominant hand located out of view under the mirror. The ball modulated up and down sinusoidally and was driven by a Clark Synthesis TST429 Platinum transducer (Clark Synthesis Inc, Highlands Ranch, CO, USA). Auditory tones were delivered through a small speaker located adjacent to the visual stimulus’s reflection on the mirror and hidden from view with black fabric. Participants made responses using a quiet, wired Apple keyboard (Apple Inc, Cupertino, CA, USA) with their non-dominant hand. The timing of all stimuli was precisely controlled using a DataPixx (VPixx Technologies, Inc) and validated using an oscilloscope.

### Procedure and Design

Each session consisted of six different modality orders, tested separately in blocks with the modality orders randomised and counterbalanced across participants. The six blocks comprised the three modality pairings (AV, VT and AT), each tested with both a positive and negative SOA during adaptation. Each block began with a prolonged adaptation phase consisting of a sequence of 180 AV, VT or AT stimuli that were asynchronous by a fixed temporal lag of either + 100 ms or –100 ms. In this study, positive SOAs always indicate a visual lead, and negative SOAs always indicate a tactile lead (meaning auditory leads correspond to negative SOAs in the AV condition, but positive SOAs in the AT condition). To avoid predictable rhythmicity, the inter-stimulus interval between successive adapting stimuli varied randomly in the range of 700 to 900 ms. Participants maintained fixation on a central white cross throughout the adaptation procedure, which lasted ∼180 s and breaks were permitted between blocks. To ensure that participants paid attention to the modality pairs during adaptation, they were asked to detect an oddball target (responding with a key-press) that could be present in either modality. More specifically, the oddball target could be a black visual ring, a louder tone or a stronger vibration that was presented in 10% of the adaptation trials.

After the final adaptation trial, a text prompt appeared for 5 s informing participants that the test phase would shortly begin. During the test phase, participants maintained fixation on the central cross and pairs of stimuli (again 50 ms in duration) were presented with various asynchronies drawn randomly from a set of pre-defined SOAs (−400, −200, −100, 100, 200 and 400 ms). Participants made a synchrony judgement in response to each test stimulus, indicating ‘synchronous’ or ‘not synchronous’ using the ‘1’ or ‘2’ keys, respectively. Each SOA was repeated 20 times within a test phase, resulting in 120 test trials in total. As in the adaptation procedure, the inter-stimulus intervals varied randomly in the range of 300 to 700 ms to avoid predictability. Each participant completed four adapt or test sessions for each of the six combinations of modality pairing and adapting asynchrony in counterbalanced order.

### Analyses

To examine the effect of prolonged adaptation on temporal recalibration, we computed the changes in PSS over time. Following a procedure used previously (Machulla, Di Luca, Froehlich, & Ernst, 2012; Van der Burg, Alais, et al., 2015), we computed a walking average of the PSS over the test period. To do so, we began by pooling the first 25 trials from each of the four test sessions of a given modality pairing and adapting SOA to obtain a sample of 100 trials and computed the proportion of synchronous judgements for each SOA. Gaussian functions were then fitted to the resulting synchrony distribution, with the mean (i.e. peak) and standard deviation (i.e. bandwidth) as free parameters. All our analyses concerned the peak of the synchrony distribution; however, it is important to note that Gaussian standard deviation, while a convenient measure of bandwidth, is often not an accurate measure of synchrony bandwidth. This is because synchrony distributions may be asymmetrical due to different perceptual criteria operating at the borders between ‘synchronous’ and ‘modality 1 first’, and between ‘synchronous’ and ‘modality 2 first’. This means that an asymmetrical distribution should be fitted to the data to properly capture the different slopes on either side of the peak of perceived synchrony, as described in (García-Pérez & Alcalá-Quintana, 2012, 2015; Yarrow, Jahn, Durant, & Arnold, 2011). As our analyses involved only the peak of the synchrony distribution (not its bandwidth or its slope on either side of the peak), we used two-parameter Gaussian functions to model the synchrony distributions.

The mean of the best-fitting Gaussian was taken as the estimate of PSS for the first 25 trials of the test phase. As there were 120 test trials, we walked the 25-trial sample window from Trials 1–25, 2–26, 3–27, … 96–120, to obtain 96 PSS estimates representing the time-course of the long-term recalibration effect. To convert from ‘trial number’ to post-adaptation duration, we computed the mean duration of a trial across sessions and observers using the time-stamps in the data files to plot the PSS time-course in terms of seconds. The mean duration for a trial was 1.65 s, and reflected three components: (a) the stimulus presentation timing, (b) participant reaction time to respond and (c) the inter-trial interval triggered by the response. The timing of Components (a) and (c) are (on average) fixed and are the largest components, but there is a small variance due to individual differences in reaction times.

To obtain the time-course of the short-term effect of inter-trial recalibration, we followed a very similar ‘walking average’ procedure (i.e. pooling the first 25 trials from each of the four test sessions). The response to each Trial (*t*) in the test phase sample was binned based on whether the SOA of the preceding Trial (*t–1*) was positive or negative. The only difference from analysing the effect of prolonged adaptation was that the analysis began with the second trial of each sample (no preceding stimulus for Trial 1), making a total sample of 4 × 24 trials. Gaussian functions were fitted to the synchrony distributions for each modality order on Trial (*t–1*), with the mean and bandwidth as free parameters. The mean of the best-fitting Gaussian was taken as the estimate of PSS for that modality order at that sample point, and by walking the sample window through the test phase, we obtained 96 PSS estimates representing the time-course of the short-term recalibration effect. This procedure was conducted separately for each participant and averaged over the group before plotting in Figures 4 and 5.

## Results

The task that participants performed to ensure they maintained attention on the stimuli during adaptation (an oddball task present on 10% of adaptation trials) was performed well. Overall mean detection accuracy exceeded 80%, validating that participants were attending to the stimuli during the adaptation phase. These data were not further analysed.

**Figure 2.**
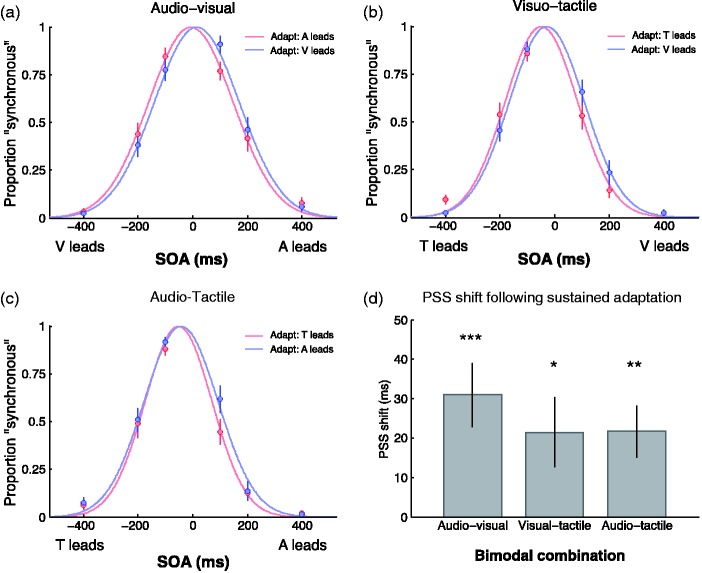
The effect of sustained adaptation on temporal recalibration. (a–c) Group mean data showing distributions of synchrony judgements as a function of SOA following prolonged adaptation to asynchronous audiovisual, visuotactile and audiotactile pairs, respectively. Adaptation consisted of a rapid sequence of 180 asynchronous stimuli whose SOA was fixed at either 100 ms or −100 ms. Temporal recalibration effects are revealed by the best-fitting Gaussian functions being shifted in the direction of the leading stimulus during adaptation. (d) Group mean PSS difference between each order of adaptation after fitting Gaussian functions to each participant’s data. Error bars show ± 1 standard error.

Figure 2 shows distributions of perceived synchrony for the three different bimodal combinations following adaptation to prolonged asynchrony. Each synchrony distribution was fitted with a Gaussian function and the mean of the best-fitting function was taken as the estimate of the PSS. The Gaussian functions in Figure 2(a) to (c) provided a good model of the data, with *r*^2 ^> .95 for all conditions. Because the test period in our experiments averaged about 180 s (during which time the recalibration effect decayed to baseline), the data in Figure 2 were taken from the first 30 s of testing while the effects of adaptation were still evident (see the time-course of recovery from adaptation in Figure 4). The recalibration effect is seen in the shift of the Gaussian functions in the direction of the leading stimulus during adaptation. In the AV condition, for example, following sustained adaptation to an AV pair with an auditory lead (i.e. negative SOA), the PSS is shifted in the negative direction, and conversely, the PSS is shifted in the positive direction following sustained adaptation to a visual lead (i.e. positive SOA).

The recalibration effects are summarised in Figure 2(d) which plots the PSS difference between both orders of adaptation (e.g. AV vs. VA) for the three different bimodal conditions. The magnitude of the PSS shifts are in the range of 20–30 ms, comparable to those reported in other studies of temporal recalibration using the conventional prolonged adaptation approach (Di Luca et al., 2009; Fujisaki et al., 2004; Navarra et al., 2007; Roseboom et al., 2015). A one-way repeated-measures ANOVA on the PSS differences was conducted to compare among the means of the three bimodal conditions. There was no significant effect of bimodal condition, with *F*(2, 57) = .519, *p* = .598, indicating that all modality combinations produced similar recalibration shifts. When compared against zero using one-tailed *t*-tests, all shifts in PSS were significantly greater than zero: AV, *t*(19) = 3.824, *p* < .001; VT, *t*(19) = 2.440, *p* < .05; and AT, *t*(19) = 3.472, *p* < .01.

**Figure 3.**
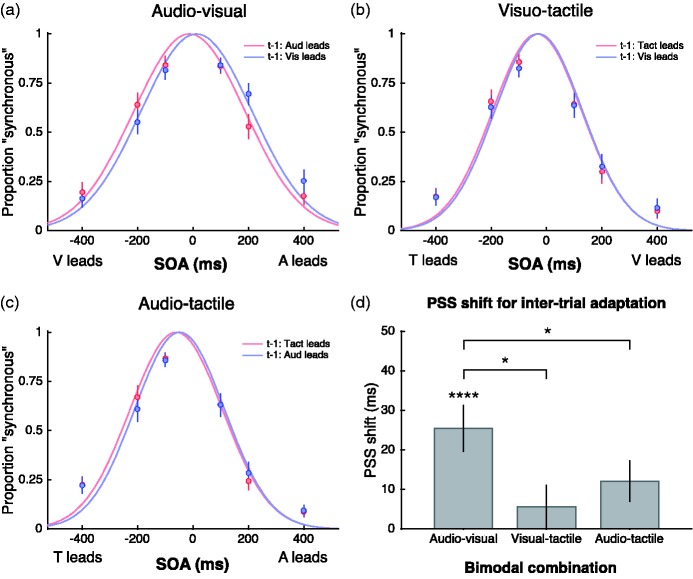
An analysis of the inter-trial temporal recalibration effect. (a–c) Group mean data showing distributions of synchrony responses as a function of SOA in the test phase that followed the prolonged adaptation period. Responses were binned into one of two categories, depending on the stimulus order in the preceding test trial (e.g. vision led or audition led). Temporal recalibration effects are revealed by the best-fitting Gaussian functions being shifted in the direction of the leading stimulus during adaptation. (d) Group mean PSS difference between each order of adaptation after fitting Gaussian functions to each participant’s data. Error bars show ± 1 standard error.

Figure 3 shows the inter-trial recalibration effect. Distributions of perceived synchrony are computed from responses to the synchrony probes presented during the test phase that followed the prolonged adaptation period. The 120 synchrony responses in a given test block were binned into two categories, depending on the modality order in the preceding trial (e.g. vision led or audition led). Each category was then sorted by SOA and fitted with a Gaussian function whose mean estimates the PSS for that category. The Gaussian functions in Figure 3(a) to (c) provided a good model of the data, with *r*^2 ^> .93 for all conditions. The difference between the PSSs within a sensory combination represents the inter-trial recalibration effect, and for all combinations, the Gaussian functions shifted in the direction of the leading stimulus on the previous trial. The recalibration shifts are summarised in Figure 3(d) which plots the PSS difference between both orders of adaptation for the three different bimodal conditions. A one-way repeated-measures ANOVA on the PSS differences was conducted to compare among the means of the three bimodal conditions. Unlike the results for sustained adaptation, there was a significant effect of bimodal condition, with *F*(2, 57) = 3.819, *p* = .028, indicating different magnitudes of recalibration shifts between the three modality combinations. Pairwise comparisons with Bonferroni correction were conducted which revealed significantly more recalibration for AV than for VT, *t*(57) = 2.701, *p* < .05, and AT, *t*(57) = 1.857, *p* < 0.05. There was no difference between VT and AT, *t*(57) = 0.844, *p* = .404.

**Figure 4.**
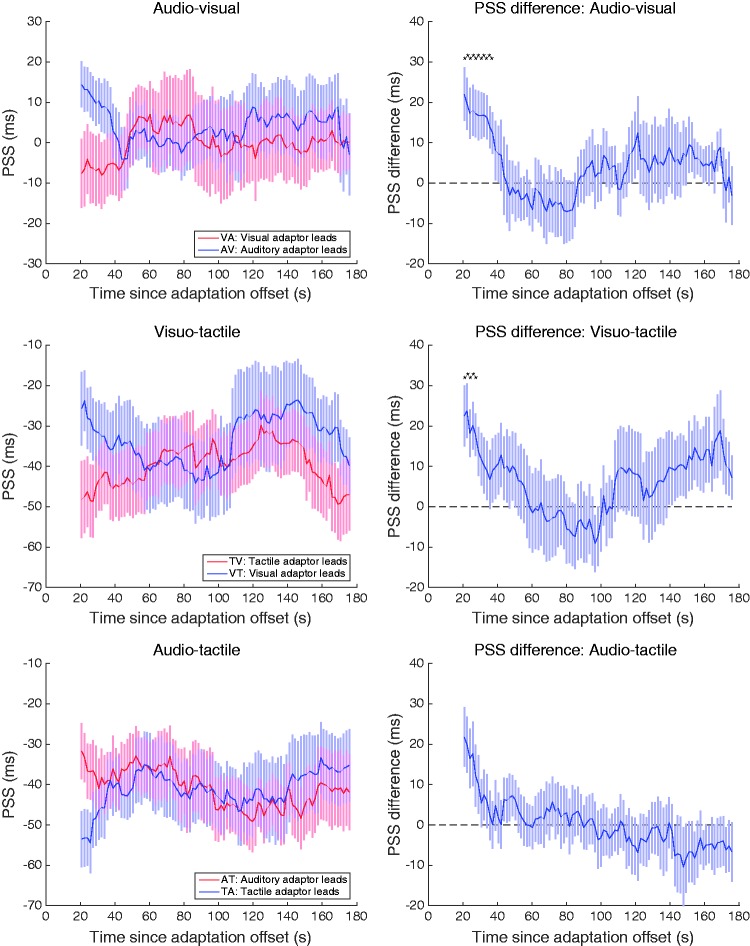
Left-hand panels show group mean data using a ‘walking average’ over a 25-trial range to show how the temporal recalibration of PSS resulting from sustained adaptation changes over time after adaptation offset, as a function of the stimulus order during adaptation. As test trials had an average duration of 1.65 s, trial numbers have been converted to time elapsed since the end of adaptation by multiplying trial number by trial duration. Right-hand panels plot the difference between the left-hand functions to illustrate the decay of the recalibration effect induced by sustained adaptation to asynchrony. Black asterisks indicate significant differences after correction for multiple comparisons using the false discovery rate method (α = .05). All error bars show ± 1 standard error of the mean.

**Figure 5.**
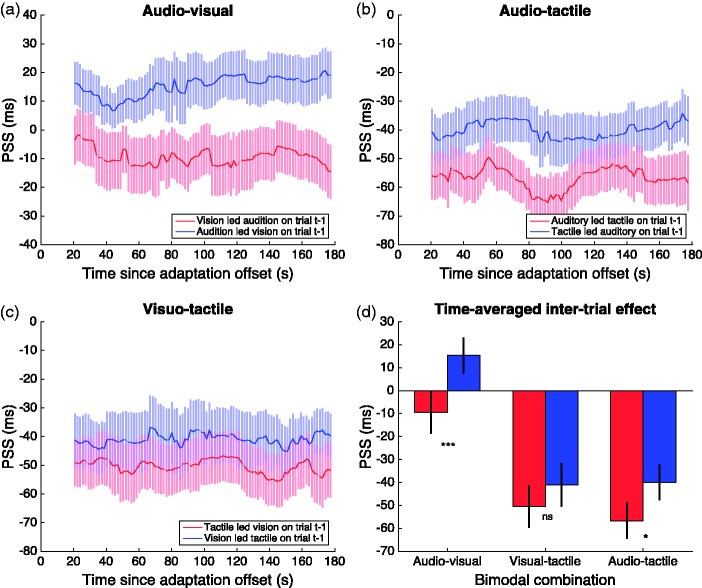
(a–c) Group mean data using a ‘walking average’ over a 25-trial range to show how the rapid recalibration of PSS changes over time since adaptation offset, as a function of the trial order on the preceding trial. (d) Mean PSS averaged across time for each sensory combination, separately for each order of adaptation. Error bars show ± 1 standard error.

When compared against zero using one-tailed *t*-tests (with Bonferroni correction, α = .05/3 = .017), the shift in PSS was significantly greater than zero for AV, *t*(19) = 4.797, *p* < .0001, while the trend for AT approached but did not reach corrected significance, *t*(19) = 2.515, *p* < .025. The PSS shift for VT was far from significant, *t*(19) = 0.970, *p* = .344.

In contrast to recalibration from sustained adaptation (present for all sensory combinations), inter-trial temporal recalibration occurs strongly for AV stimuli, is absent for VT stimuli and is present but weaker for AT stimuli. The magnitude of the AV PSS shift (∼25 ms) shown here for inter-trial recalibration is comparable to magnitude reported earlier following sustained adaptation.

The recalibration effects shown in Figure 2 following sustained adaptation can be plotted as a function of time using a walking-average approach (as described in the Methods: see Analyses subsection) to reveal the time-course of temporal recalibration following offset of the sustained adaptation (Machulla et al., 2012; Van der Burg, Alais, et al., 2015). This walking-average approach produced a total of 96 PSSs at 96 different time points (i.e. from Trials 1–25 to 96–120) and reveals the initial magnitude of temporal recalibration and the time-course of its recovery. Group mean PSS data obtained with this approach with ± 1 standard error bars are plotted in Figure 4 for each of the three modality combinations. The PSS data are plotted as a function of time elapsed since the end of adaptation, calculated on the basis that each test trial took an average of 1.65 s to complete (group mean time to complete 120 test trials was 198.5 s).

The plots on the left-hand side of Figure 4 show that all bimodal combinations produced a strong initial recalibration following sustained adaptation. For all three modality combinations, the PSSs were initially shifted in the direction of the leading stimulus during the extended adaptation period (the plots show data for both adaptation orders overlaid). To examine the time-course more clearly, the right-hand panels plot the difference between the functions in the corresponding left-hand panels. The magnitudes of the initial recalibration effects in the difference data appear remarkably similar (AV = 20 ms, VT = 21 ms and AT = 19 ms), an average of approximately 20 ms over the first few bins in all three bimodal combinations, before all functions converge towards baseline over time. This indicates that the time-course of recalibration following sustained adaptation behaves similarly across all modality pairings; however, to examine further the significant effect of post-adaptation time, specifically its return to baseline, we tested all time points within the first 60 s to see which were significantly greater than zero, correcting for multiple comparisons using the false discovery rate (FDR) method (α = .05). The asterisks on the difference plots in Figure 4 indicate significant elevations above zero after FDR correction. This point-wise analysis of the early post-adaptation period – where the recalibration effects are concentrated – suggests there are recalibration differences between modality pairs following sustained adaptation. The AV condition clearly produces the longest effect, with the first 11 time points being significantly above zero after FDR correction. In comparison, only the first four time points were significantly above zero in the VT condition. The effect was weakest in the AT condition, with none of the points being significantly above zero after FDR correction was applied.

Next, we conducted the same time-course analysis on the inter-trial effect (again with a ‘walking average’ over a range of 25 trials) to see how the magnitude of rapid recalibration varies throughout the test period. The results are plotted in Figure 5 and show very clearly that temporal recalibration arising from inter-trial adaptation is strikingly consistent throughout the test period. This was shown recently for inter-trial recalibration to AV asynchrony (Van der Burg, Alais, et al., 2015). Here, we replicate that effect for AV stimuli and extend it to AT inter-trial recalibration. There was no significant recalibration for VT asynchrony at any time point. These plots reveal consistent effect sizes throughout the test period that match the distributions shifts plotted in Figure 3. The time-averaged PSS shifts are summarised in Figure 5(d) and paired *t*-tests (two-tailed) show a very strong inter-trial recalibration for the AV condition, *t*(19) = 4.761, *p* < .0001; no effect for the VT condition, *t*(19) = 1.338, *p* = .197; and a significant effect for AT, *t*(19) = 2.223, *p* < .05.

## General Discussion

The present experiments compared cross-modal temporal recalibration from sustained and transient adaptation in three different sensory pairings: AV, VT and AT. Our design enabled us to compare the effects of both brief and sustained adaptation within a single experiment, using the same observers, stimuli and procedure in all three sensory combinations, and with all stimulus components spatially collocated. Our results confirm previous studies that recalibration following sustained adaptation occurs for all three sensory pairings and show equivalently strong recalibration for all combinations. However, under the same conditions, recalibration from inter-trial adaptation was present for AV and AT but not for VT. In the inter-trial conditions, the AV condition stood out as exhibiting the strongest recalibration effect. For all three sensory pairings, recalibration from sustained adaptation was found to be independent of inter-trial recalibration, consistent with separate mechanisms underlying the two types of recalibration.

### Prolonged Adaptation

Our findings for recalibration from sustained adaptation confirm existing reports using AV stimuli and clarify a discrepancy among others using AT and VT stimuli. Our AV results are comparable to several other studies that used prolonged adaptation to induce temporal recalibration of AV stimuli in showing shifts in PSS of around 30 ms in the direction of the stimulus component that led during adaptation (Fujisaki et al., 2004; Navarra et al., 2005; Roseboom & Arnold, 2011; Vatakis et al., 2007; Vroomen et al., 2004; Yarrow, Roseboom, et al., 2011). Notably, some of these studies used spatially collocated AV stimuli (Di Luca et al., 2009; Yarrow, Roseboom, et al., 2011) and others did not (Fujisaki et al., 2004; Navarra et al., 2007; Roseboom et al., 2015), yet results are comparable regardless of the spatial configuration of the component stimuli. This independency implies a temporal recalibration process for AV that is purely temporal and occurs regardless of the spatial location of the components (Fujisaki et al., 2004; Keetels & Vroomen, 2007). As depicted in Figure 2, our results also show that recalibration of PSS is possible for AT and VT combinations (here using collocated stimuli) following prolonged adaptation, with no statistically significant differences in recalibration magnitude among the bimodal conditions evident in our data.

Spatial collocation of the stimulus components appears to be a relevant factor for recalibration of AT and VT stimuli because previous studies using prolonged AT and VT adaptation have produced mixed results. Generally, previous studies have found PSS shifts after prolonged exposure to asynchronous AT and VT stimuli (Hanson et al., 2008; Keetels & Vroomen, 2008; Takahashi et al., 2008), although different results may be obtained depending on the spatial disparities between stimulus pairs. For example, adaptation to AV asynchrony was found to transfer to produce recalibration in AT stimuli when the stimuli were external and spatially aligned but did not transfer when the sounds were presented through headphones and therefore spatially decoupled (Di Luca et al., 2009). While other studies have used collocated VT stimuli yet did not find recalibration (Harrar & Harris, 2005, 2008), this failure probably reflects the fact that VT adaptation decays quickly and the frequency of top-up adaptation was insufficient (Ho, Orchard-Mills, & Alais, 2015). Although our experiment does not manipulate spatial location, our study is the first to compare all bimodal conditions under matched experimental conditions using the same observers in all conditions and with all component stimuli spatially collocated. Our results therefore confirm that AT and VT recalibration do occur following sustained adaptation with spatially collocated components.

As well as providing a matched comparison of recalibration from sustained adaptation across three sensory pairings, the method used here allows us to plot the time-course of recalibration following offset of adaptation. The results plotted in Figure 4 (left-hand panels) show that the patterns for each sensory pairing are qualitatively similar: initially, there is a large recalibration shift of PSS towards the modality that led during adaptation, followed by a convergence towards a neutral baseline. The initial magnitudes of PSS shift were virtually identical across the three sensory pairings, indeed all fell within 1 ms of each other. For the first time-bin shown in Figure 4 (difference data: right-hand panels) the differences were AV = 22 ms, VT = 23 ms and AT = 22 ms, and averaging over the first three bins produces an average recalibration of 20 ms for all combinations. However, the rate of convergence towards baseline differed among the three sensory pairings. Using 95% confidence intervals to determine which of the initial bins show a difference greater than zero, the first 11 bins are significant for AV, the first 6 for VT and the first 4 for AT. With each bin representing 1.65 s, and the first bin being at 20.63 s (mean of the first 25 trials), the total duration required for recalibration to recover to baseline for each sensory pairing is as follows: AV = 37.1 s; VT = 28.9 s; and AT = 25.6 s. Moreover, the rate of decline over the significant initial bins varies considerably among the three pairings: AV = 1.33 ms/,; VT = 2.79 ms/s and AT = 4.44 ms/s. The time-course data therefore reveal that while the initial magnitudes of recalibration are equivalent in all conditions, the three sensory pairings have different recovery rates. The AV condition stands out as having the longest-lasting effect by being the slowest to recover to baseline, being approximately 2 × slower than VT recovery and 3 × slower than AT recovery.

### Inter-Trial Adaptation

Significant inter-trial recalibration was observed for the AV and AT conditions but not for VT. The inter-trial data show a very different pattern compared to the data from sustained recalibration in that a large recalibration is evident from the beginning of the test period and it is maintained throughout testing with no decline in magnitude (cf. Figures 4 and 5). For the AV combination, both the recalibration magnitude and its sustained pattern are consistent with our earlier reports conducted with non-collocated stimuli (Van der Burg et al., 2013; Van der Burg, Alais, et al., 2015). The current results complement those earlier reports in showing that the effect also occurs when stimuli are collocated, establishing that inter-trial recalibration for AV stimuli occurs without spatial selectivity. Although the AT combination also produced significant sustained and inter-trial recalibration, it was significantly weaker than for the AV stimuli, as is clear from Figures 4 and 5. These data, then, confirm our previous result that inter-trial AV recalibration is unique, in that it is strongest effect (see Figure 3) and occurs regardless of the spatial relationship of the components (cf. Van der Burg et al., 2013).

The finding of inter-trial recalibration for AT stimuli is a new result that was not observed in our previous study (Van der Burg, Orchard-Mills, et al., 2015). There are two potential explanations for this, one being that the effect was not detected in our previous experiment (i.e. a Type-II error) due to the magnitude of the AT effect being smaller than the AV effect (see Figure 3), the other being that the effect requires spatially collocated stimuli, which was true for the current experiment but not the previous one. Given the high statistical power of the earlier study’s data (Van der Burg, Orchard-Mills, et al., 2015), a Type-II error is improbable and a requirement for spatial collocation for AT recalibration appears more likely. This raises the question of why there would be spatial selectivity for AT but not for AV? One reason would be that because a tactile stimulus must directly impinge on the skin there is no (external) distance dependency and while internal transmission times to the brain from various parts of the body differ, they are largely constant for a given body location and can easily be learned and discounted without the need for a dynamic, transient recalibration. Indeed, because the variance in tactile latencies is largely internal, it would be maladaptive to recalibrate from moment to moment as it would in effect misrepresent the physical timing of stimuli on the body. Given this risk, it is not unreasonable that rapid, inter-trial recalibration between A and T components should require a spatial collocation condition to be met as this condition is consistent with sounds being causally related to the pressure or vibration on the skin.

The final conclusion from the inter-trial data is confirmation that there is no inter-trial recalibration for VT combinations, when neither collocated (see Figures 3 and 5) nor non-collocated (Van der Burg, Orchard-Mills, et al., 2015). The confirmation here of our previous null result for VT stimuli is important, as the current experiment maximised the chance of detecting a rapid VT recalibration by using collocated stimuli and using the same observers, stimuli and procedures. The failure to find rapid VT recalibration in these experiments is telling because here all three sensory pairings were examined in a matched paradigm so the null result cannot be attributed to procedural differences. Under these matched conditions, only the VT pairing failed to produce inter-trial recalibration. Moreover, the same V and T stimulus components did produce inter-trial recalibration in other combinations and so it is unlikely to be stimulus related. The lack of inter-trial VT recalibration probably reflects the fact that most latency variability between V and T stimuli is internal and thus predictable and can be learned and discounted. Our data support the conclusion that only sensory pairings with an auditory component – AV and AT – exhibit rapid recalibration because only those conditions have to deal with variable auditory latencies arising externally, namely, sound source distance.

### Implications

An interesting distinction is that VT recalibration does occur following sustained adaptation but shows no inter-trial recalibration. This difference is not a spurious one as the lack of inter-trial effect for VT reported here (Figures 3 and 5) adds to our previous failure to find inter-trial VT recalibration (Van der Burg, Orchard-Mills, et al., 2015), whereas here (Figures 2 and 4) and elsewhere it has been shown that sustained adaptation does produce VT recalibration (Hanson et al., 2008; Ho et al., 2015; Keetels & Vroomen, 2008). We suggest two possible accounts for this. One would be that sustained adaptation and inter-trial adaptation engage separate recalibration processes (a two-stage model), with sustained adaptation effecting gradual change in a sluggish process (such as updating a Bayesian prior distribution of temporal asynchronies, which would be useful for any of the three sensory combinations), and inter-trial adaptation engaging a transient recalibration process whose purpose is to deal with short-term variability in cross-sensory timing when auditory components are involved. Under this dual-level framework, there would be no VT effect because transient recalibration would be limited to AV and AT by definition.

Another way to account for the absence of VT recalibration in the inter-trial paradigm would be in terms of the sensitivity of a single-level process. On this account, adaptation would occur very sluggishly for VT, producing smaller effects even following sustained adaptation and none at all following transient adaptation. This proposal is attractive theoretically in that it requires only a single process and is also consistent with the data: The VT condition produced the smallest effect size following sustained adaptation (Figure 2(d)). If this is evidence of a more sluggish process for VT than for AT and AV, then it would be expected to show less effect following transient adaptation. Consistent with this, VT also produces the smallest (and non-significant) effect size for inter-trial recalibration (Figure 3).

Neither of these accounts can explain all the data in all three sensory pairings. The ‘single-stage sensitivity’ account, for example, explains the weaker recalibration for VT following short-term relative to long-term adaptation but cannot explain the AV and AT results. For those sensory combinations, adaptation from a single trial produced as much recalibration as 3 min of sustained adaptation (cf. Figures 4 and 5), and this is clearly incompatible with a gradual accumulation of adaptation in a single mechanism. Both accounts can be subsumed in a two-stage model in which the sluggish temporal recalibration process implied by the VT data exists for all sensory combinations, and a second transient process exists for dealing with combinations containing an auditory component (AV and AT only). Consistent with this proposal, AV and AT combinations both showed significant recalibration for sustained and for transient adaptation, yet we found no statistical interaction between these factors, implying that they are separate and additive components. Moreover, evidence suggests the transient recalibration effect is inherently a process for dealing with relative timing of multisensory signals as inter-trial recalibration is not observed between two auditory or two visual stimuli (Harvey, Van der Burg, & Alais, 2014).
